# On Glanders and Farcy in Man

**Published:** 1838-07

**Authors:** 


### Art. VIII.

*De la Morve et du Farcin chez l'Homme.* Par P. Rayer, m.d., Médecin de l'Hôpital de la Charité, &c. (*Mémoires de l'Academie de Médecine,* tome vi.)—*Paris,* 1837. 4to. pp. 878.

*On Glanders and Farcy in Man.* By P. Rayer, m.d. &c.

It is at once instructive and interesting to follow the gradual and steady progress from surmise to conviction, from a single vague fact to the imposing mass of evidence in the volume that lies before us, respecting the possible transmission of the poison of glanders from the horse to the human subject. We need, therefore, offer no apology for indulging in a tolerably full abstract of the well-digested historical survey with which that volume opens.

The earliest hint on the question of such transmission is said by M. Rayer to be contained in a work by Waldinger, published in 1810, in which the author enjoins the strictest caution in opening the bodies of glandered horses, as the most deplorable consequences, even death itself, may ensue, if the inoculation of pus from the diseased animal be allowed to take place through an abraded surface. In 1812, Lorin, a French surgeon, published the case of an individual who suffered under severe inflammation of the fingers of both hands, in consequence of a prick received in operating on a horse affected with button farcy. Observations resembling these, which allude simply either to local inflammations or affections similar to those resulting from the absorption of matter in ordinary dissection-wounds, had been made in various quarters, when (in 1821) Schilling's well-known case appeared in Rust's Magazine. In this case, designated by Rayer—justly, as it appears to us, though its nature is still a matter of doubt for some,—as "the first distinct and well-characterized instance of acute gangrenous glanders in man," the evidences of specific infection consisted in the peculiar glanderous discharge from the nostrils, a pustular cutaneous eruption, and a gangrenous state of the nasal integuments. In the Edinburgh Medical and Surgical Journal, for April, 1822, is to be found a brief and unsatisfactory report of the case of a man, who wounded his hand in cutting up a glandered horse, and shortly after presented all the symptoms of the disease, as observed in the horse: at the end of a week, he is stated to have died in violent delirium of "*confirmed glanders.*" The Number for January, 1823, of the same Journal, contains a letter from an anonymous London physician to Dr. Duncan, in which allusion is made to some important facts relating to the subject before us. The writer refers, as to a well-known circumstance, to the malady of a veterinary surgeon, that had recently terminated fatally, having been "contracted from a farcy horse, and characterized by ulceration in the part of the arm infected, and by what are called farcy buds extending up the arm." And, further on, he informs his correspondent that there had lately been a patient, at one of the London hospitals, with a sore arm, resulting from a wound and contact with the leg of a farcied horse; that an ass was inoculated with matter taken from the patient's arm; that farcy, followed by glanders, ensued; and that, on the animal's death, ulceration of the septum narium was detected. In 1822, Remer, in an essay on the Diseases of Animals communicable to the Human Being, asserted that "the glanders of horses infects men." In the same year, an Italian practitioner, Tarozzi, published an account of a pestilential disease that originated in a stable where a glandered horse was kept. Of thirty-five individuals who visited the stable frequently, eleven were seized with an affection characterized by fever, and an eruption of boils and gangrenous phlyctenæ. No allusion is made in the description, however, to a morbid condition of the nares.

The next contribution of importance appears in Mr. Travers's essay on Constitutional Irritation. The work and its doctrines are so well known that we should consider it unnecessary either to notice the facts it contains bearing on our question or the author's deductions therefrom, were it not that M. Rayer has very clearly mistaken the nature and scope of the latter, and given Mr. Travers credit for opinions other than those he maintains. In the cases observed by Messrs. Travers and Coleman, affections possessing the characters of farcy and glanders were distinctly communicated from diseased animals to the human subject; and, on inoculation of the pus secreted by the latter into healthy asses, the specific disease made its appearance, and terminated fatally in the animals experimented on. M. Rayer naturally infers that, on evidence so conclusive as this, Mr. Travers must have admitted the possibility of the transmission of glanders to man: but the fact is otherwise. Mr. Travers, in spite of what he had seen, declares that "there is no evidence of the poison of glanders acting otherwise on the human body than as the poison of dead animal bodies." And, in order to reconcile his experience with the preconceived theoretical notion to which he evidently accords the preference, he has recourse to the extravagant hypothesis that the *specific* property of the poison is retained in its passage through the human system, though incapable of specifically affecting it!

The Medical Gazette for July, 1829, supplies the next valuable document in order of priority, in the form of a well-marked case of glanders, observed by Mr. Andrew Brown. The prominent features of the case were as follows:—Contact with a glandered horse; appearance of cutaneous pustules terminating in gangrene, and of tumours round the joints; "the right nostril contracted, and gummed with an inspissated discharge;" after death, "well-defined ulcerated tubercles discovered in the Schneiderian membrane, exactly the same as those of acute glanders in the horse;" pus in the muscles, under the skin, and in the frontal sinuses, &c. Mr. Brown regards his case as of importance in regard of a question first (we believe) mooted by him; namely, whether the disease be transmissible without the aid of an abraded surface.

This series of facts, joined to many others which we have no space to notice, was sufficient for forming the groundwork of a moderately complete history of the disease in the human subject, and, at least, for placing the reality of the specific transmission beyond the reach of doubt. Strange to say, however, they aroused little attention, and pro duced no conviction; nor was it until the appearance of Dr. Elliotson's paper in the Medico-Chirurgical Transactions (read in June, 1830,) that the subject received the notice due to its importance. In this respect M. Rayer does full justice to the deserts of our countryman, and he acknowledges that to Dr. Elliotson he owed his first notion of the disease. Besides supplying three additional and very distinct cases of the affection, the paper alluded to possesses the sterling merit of having made public what had previously been known only to the few. But, so far as we can ascertain, it contains no then-unnoticed fact bearing on the subject; while, it must be admitted, its cases are particularly valuable, as they were observed and registered without any preconceived notion as to their character on the part of their author. So inexplicable, indeed, did their phenomena appear to him at the time of their occurrence, that, when asked for his opinion of their nature, he "declared himself perfectly at a loss." The publication of Mr. Andrew Brown's case enabled him, as he avers, to clear up the diagnosis, and convinced him that the novel affection he had observed was none other than glanders.

From this period cases and papers multiplied in German and English journals. Of all these M. Rayer has shown himself a faithful chronicler. They have now accumulated to such an extent that it is hardly just to consider the disease a *rare* one absolutely, much less relatively speaking, —that is, if we pay due regard to the limited number of the individuals whose position exposes them to contagion. Dr. Graves, in a Lecture recently published, maintains it to be so common an affection in the provincial parts of Ireland, that, in his opinion, the legislature should, in imitation of the Prussian authorities, subject glandered animals to official supervision.

The author begins the proper subject of his essay by distinguishing in man, as in the Solipeda, three varieties of the acute form of the affection; the pustular, the gangrenous, and the pustulo-gangrenous.

"In some cases, the most striking phenomena consist of a pustular cutaneous eruption, a thick and glutinous nasal discharge, and a typhoid aspect. In others, the symptoms of nasal lesion are obscure, while the external characteristics (the pustular eruption and gangrenous affection of the skin) predominate. Again, pains in the limbs, purulent deposition in various parts of the body, and inflammation of the lymphatic vessels and glands, form, in the outset, the most striking features of another set of cases, still more strictly analogous in character to the acute farcy glanders of the horse. Lastly, there are others more violent and promptly fatal, in which all these morbid changes and symptoms appear rapidly and at once, a few days after vague initiatory phenomena." (p. 642.)

But the classification adopted in the arrangement of his collected cases is founded on another, and perhaps more important, view of the subject, —the causation of the disease by inoculation, or, as all appearances seem to indicate, by infection without contact. The question of simple infection, as we have already stated, was touched on by Mr. Brown; and the case so diligently observed by that gentleman was of a kind fully to warrant his evident, though not professed, belief that his patient had been the victim of infection. He informs us that "the absorbent vessels of both arms, as well as their glands, were found in the natural state; nor was there the slightest appearance of either absorbent, glandular, or cutaneous inflammation; nor the slightest breach of integument or abrasion of skin by which the absorption of morbid matter into the system could have been facilitated." The first part of this statement contains the distinctive character of a "class of cases in which no local results of inoculation are observed; the first symptoms being those of a general infection of the system. In another, the earliest phenomena are those of angeioleucitis, local phlebitis, or diffuse cellular inflammation in some region of the body, usually the arm; while the lesions and symptoms of *glanders* do not appear until subsequently." (*Rayer*, p. 643.) This broad distinction in the phenomena observed, coupled with the fact that, in cases of the first class, the closest scrutiny failed in detecting any injury of the skin, constitutes M. Rayer's reason for admitting, provisionally at least, the transmission of the disease to man by simple infection. The English reader, prepared as he has been for it by the surmise of Mr. Brown and others, will, on the adduced evidence, probably accede to this conclusion without much difficulty. To the countrymen of the author, however, it must appear somewhat startling; at least, unless their notions on this and analogous subjects have undergone much modification since 1832, when Parent-Duchatelet asserted that "none of the supposed infectious diseases of animals exercised an unfavorable influence on the health of man."*

For our own parts, we place more confidence in the natural and clear symptomatic distinction of the cases into two classes; the primitively local, and those in which, from the first, the whole frame is affected, than in the absence of discernible breach of surface as indicating the reality of infection. Dr. Elliotson was of opinion that "abrasion of the surface is necessary to the production" of the malady, basing his belief on the notion then (1830) prevailing among the majority of veterinary surgeons, that "glanderous matter never excites the disease in the horse, even if applied to the pituitary membrane, while the surface is entire:" but this opinion is no longer general. In a recent and valuable paper, Dr. Eck, of Berlin, expresses himself thus: "And our medical police regulations assume as proved the contagious nature of glanders; a fact, indeed, of which experienced horse-dealers scarcely anywhere entertain a doubt. Now, though the glanderous nasal discharge is recognized as the chief vehicle of infection, and, on this account, the use of harness, mangers, drinking vessels, &c., which have been in contact with glandered horses, and are liable to be brought in contact with the pituitary membrane of sound animals, is especially to be feared; yet it can hardly be doubted that the contagion may exist in the other excretions; nay, that occasionally, as in damp stalls, infection may take place without any observable contact, probably through the medium of the respired air."†

Our author has collected nine examples of the disease resulting from infection. Of these the first alone is original, and supplied the "motive and the cue" for the compilation of the present bulky treatise. For the details of this case, which occupy twenty pages, we must refer the reader to the original. The chief points in the history may be briefly given

* Gazette Médicale, Déc. 1832, t. iii. No. 118.

† Medicinische Zeitung, Mai 3, 1837. No. 18, s. 85.

thus: A groom, who slept in a stable near a mare affected with acute farcy glanders, is seized with typhoid symptoms, subcutaneous abscesses, pustular and gangrenous eruption of the skin, nasal fossæ, and larynx; ecchymoses and eschars below the ear, on the glans penis and feet; small abscesses in the legs, large subcutaneous and muscular depositions of pus, &c. The fluid from the pustules and phlyctenæ produced glanders in a horse by inoculation. The other cases originating, according to Rayer, in simple infection are those reported by Brown, M'Donnell, Elliotson, Remer, Wolff, Alexander, and Schilling. In two of these, mention is made of a slight scratch. Six cases of acute glanders, produced by inoculation, follow, conducting us to the author's summary on this form of the disease.

In addition to the resemblance as to cause, course and duration, that subsists between acute glanders in man and affections produced by the absorption of pus or morbid poisons, it would appear that there occurs in this disease, as in eruptive fevers, a variable though limited period of incubation. Thus, after inoculation of glanderous matter, a period of from two to eight days, or more, elapses before the occurrence of any notable symptoms. At the end of this time the evidences of local irritation appear at the injured part; its extension follows, as is proved by the condition of the neighbouring lymphatic glands and vessels, and by general febrile phenomena. In some instances these local symptoms were slight, and easily relieved; the patients even appeared on the brink of recovery, when the essential and specific symptoms of glanders supervened. When the disease is contracted by *infection*, the invasion is marked by fever, attended with gastric symptoms, diarrhœa, or pains in the limbs.

Ushered in in this manner, the disease ordinarily presents the following phenomena in its course:—Articular or muscular pains, in some cases simulating rheumatism, followed by subcutaneous, circumscribed, painful swellings (the probable result of angeioleucitis), which either undergo superficial mortification or are converted into abscesses, containing laudable or sanious pus; a yellowish, viscous, nasal discharge, of limited quantity, issuing, in the great majority of cases, from *both* nares, and first observed from the fourth to the sixteenth day, accompanied in some instances by a similar excretion from the mouth or eyelids; occasional tumefaction of the nose and adjoining parts, followed by gangrene in one-eighth of the cases; in very rare examples, swelling of the submaxillary lymphatic glands, or depositions of pus therein; and pretty frequently inflammation of the throat and tonsils; a peculiar pustular eruption, differing from all varieties hitherto observed,—viz. gangrenous bullæ appearing, towards the twelfth day of infection, on the face, arms, thighs, and anterior surface of the trunk, and sometimes preceded or accompanied by profuse fetid sweats; rapid and full pulse at the outset, subsequently weak, depressible, and sometimes intermittent, and, as death approaches, extremely small and frequent; diarrhœa, with watery stools of cadaverous smell, and occasionally containing dark-coloured blood; dental sordes; dry, brown tongue; tympanitic abdomen, with hardly any abdominal tenderness; thirst in a few cases; difficult deglutition; occasional vomiting, especially towards the close; *no typhoid*

*maculæ* on the surface; violent cerebral symptoms, terminating in delirium, coma, and death.

As respects the mortality of the disease, M. Rayer states:

"Acute glanders in the human being has hitherto invariably terminated in death, (one doubtful case excepted,) and two-thirds of the patients perished before the seventeenth day. Two died from the twenty-first to the twenty-eighth day; one only survived to the fifty-ninth. In the latter case, the early symptoms were those of farcy; those of acute glanders supervening as the final phenomena, in the same manner as has sometimes been observed in horses." (p. 728.)

In analysing the results of the recorded examinations after death, M. Rayer found two obstacles in the way of accurate conclusions,—the imperfect manner in which the investigation was almost always conducted, and the use, by different writers, of the same word in different senses. Thus, the various significations which the term "tubercle" is made to assume in this country, according to the whim of authors, are productive of much confusion among ourselves; while to a foreigner, accustomed to limit its application to the anatomical character of phthisis and to a particular class of cutaneous affections, this inaccuracy of language renders much otherwise valuable matter completely unintelligible. Spite of these difficulties, the following results are valuable:—Externally traces of the pustular eruption are constantly observed, besides, in the majority of cases, gangrenous bullæ or cutaneous sloughs. The nares presented lesions eminently characteristic of the disease in the only four cases in which they were examined; these were ecchymoses and gangrene of the pituitary membrane, minute abscesses, ulcerations of the septum, granulations in the frontal sinuses, or thickening and infiltration of the pituitary membrane, which was studded with ulcerated "tubercles." The larynx, rarely examined, is however noted as affected with a peculiar eruption in the author's own case, with ulceration or inflammation in others. Of the condition of the trachea little is known. In the fourteen fatal cases, the lungs were not examined four times; in three instances, they were declared healthy. In the remainder, lobular pneumonia, pleuro-pneumonia, "vomica," pleural tubercles with abscess, dark-coloured sanguineous engorgement of the pulmonary tissue, existed in various degrees. The blood, occasionally buffed at the outset, was liquid and of slight consistence towards the close or after death. The heart and great vessels were healthy. The lesions described as occurring in the alimentary canal were slight in degree, and by no means constant in appearance; besides, the vague language used in their description renders it impossible to form any correct opinion of their nature. In the subcutaneous and intermuscular cellular membrane were found collections of white gelatinous pus, infiltrated serum, purulent depositions under the periosteum of the cranial and shoulder bones.

The differential diagnosis of the affection has evidently been studied with much care by M. Rayer; but the importance of his results does not justify the extreme diffuseness in which he indulges. They amount in the main to this: that acute glanders may be distinguished from the results of dissection-wounds, of absorption of pus after capital operations, from ordinary phlebitis, angeioleucitis, malignant pustule, carbuncular affections, putrid variola, &c., by the peculiar nasal discharge, pustular eruption, and the property the secreted pus possesses of producing the specific disease in sound animals. The nice points of distinction brought into notice by the author are, however, we admit, worthy of study.

An elaborate description of the disease as observed in the horse, and founded on M. Rayer's own researches, is next given, in proof of its identity with the human malady. We shall allow the curiosity of our readers to seek this in the original, and pass at once to the remarks on treatment, which, indeed, contain little worthy of extraction.

If, when first called in, the medical attendant find the lymphatic vessels and glands inflamed, the author recommends the immediate excision of the swollen glands; and, if the local inflammation following this operation, or resulting from the inoculation alone, were intense, "débridement" and mercurial frictions might, he presumes, be employed with advantage. He is decidedly hostile to general or topical blood-letting, notwithstanding the buffy condition of the blood in the early stage: if the former be employed, prostration and stupor rapidly supervene; if the latter, local gangrene. The use of bark, serpentaria, and other tonic antiseptics, is declared to be equally fruitless. The treatment to which he gives the preference consists in repeated purgation and the exhibition of large doses of acetate of ammonia; a favorite remedy with veterinary surgeons. Whatever be the number of abscesses formed, they should be incised, and the cutaneous pustules and bullæ opened and cauterized; while the patient's strength is supported by "tonic drinks and generous wine diluted with a gaseous water."

There seems, from these statements, to be but a sorry chance of devising, by the aid of experience hitherto acquired, any feasible plan of arresting the progress of this frightful disease. In the cases collected by M. Rayer, especially the German ones, every variety of rational treatment appears to have been employed with vigour and discernment, yet those in charge of the patients had not the satisfaction of indisputably checking for a moment the violence of the symptoms or the rapid course of the malady. If such be the truth, (and the cases themselves prove it to be so,) the fair inference therefrom seems to be, that the wisest plan, —at least as regards *general*, in contradistinction to *local* treatment,— would be to trust mainly to the powers of nature. The remarkable form of disease from which Professor Roux recovered under treatment almost purely expectant, (a few applications of leeches being the only active means employed,) bearing, as it did, a very strong analogy to glanders in many of its characters, seems to justify a trial of the mildest therapeutic measures in the latter disease, in preference to a repetition of the heroic remedies that have hitherto so signally failed.

In his account of *button farcy*, M. Rayer agrees with the majority of those who have written on the subject, in viewing that form of affection as produced by the same contagion as glanders, and differing solely from this in the situation of the affected parts: and, as he very justly remarks, some of the fifteen cases that follow, though quoted as examples of simple farcy, may, in reality, have been instances of farcy glanders; for, though no nasal discharge was noted during life, yet (inasmuch as the nasal fossæ were not examined after death) it cannot be affirmed that they were free from glanderous eruption. Exposure to contagion is pretty evidently made out in these cases. In four of them the disease was evidently inoculated; in the others, the mode in which the contagion acted was not ascertained.

When the affection results from inoculation, the first symptoms are those of angeioleucitis, attended by diffuse cellular inflammation. If the spot where the morbid matter was introduced suppurate, a true pustule is occasionally formed, succeeded by a foul ulcer. The inflammation of the superficial lymphatics is sometimes sufficiently great to produce real farcy cord. These local derangements are accompanied by fever, anorexia, nausea, shivering, &c. So far, however, the occurrence cannot be distinguished by its effects from an ordinary dissection-wound; their cause alone distinguishes them. If the glanderous poison enter no further into the system, recovery frequently takes place. If otherwise, the characteristic pustulo-gangrenous eruption and glandular abscesses supervene, announce a general infection of the blood, and are, with rare exceptions, followed by death. The specific eruption varies as to the time of its appearance, from the fourth day to the third or fourth week after inoculation. The eruption is preceded or accompanied by copious sweats, and occasionally by gangrene of the cheeks or other parts of the body. Add to this the appearance on the limbs, and elsewhere, of soft doughy tumours, small and slightly prominent, usually almost indolent, and very generally ending in suppuration or gangrene. These tumours, however, possess no character distinguishing them from those of ordinary angeioleucitis. Death, which almost always takes place if there be glanderous eruption, supervened from the thirteenth to the nineteenth day, preceded by prostration, delirium, involuntary fetid stools, &c.

But, as the reader will probably enquire, if the non-eruptive form of the disease be completely similar in its symptoms to the affection resulting from ordinary dissecting wounds, can it fairly be ascribed to a specific farcy infection? In the absence of the only information which would justify a positive conclusion,—namely, the results of inoculation of the horse or ass with the pus secreted by the human subject,—we confess ourselves little inclined for a discussion of the question. M. Rayer, in adopting the affirmative, appears to have lost sight of the prudent reserve that guides him in the majority of his conclusions. It may be, and we believe it is, true that "circumscribed angeioleucitis and successive formation of abscesses have been more frequently observed to follow anatomical wounds in veterinary than in medical practitioners;" but we should be sorry to found our opinions on evidence so indirect and unsatisfactory. If the non-eruptive disease be really a variety of farcy, it at least differs materially from the acknowledged form of the affection, as it does in the mortality it occasions. Of five cases without eruption, four terminated favorably, after one or more months' suffering, it is true; while nine of the eleven subjects in whom the eruption existed succumbed. Our author's paragraphs on the appearances after death are of necessity meager in detail, from the careless and incomplete manner in which the examinations were made: those on diagnosis are a repetition of facts and opinions already noticed: those on treatment exhibit nothing novel.

A lengthy chapter follows containing reports of "cases of doubtful nature." There are valuable materials in these for substantiating the diagnosis of analogous affections. M. Roux's account of the malady he contracted from a wound of the left index finger, while deeply inserted into the rectum of a patient on whom he was operating for fistula in ano, will, in spite of its egotistical pompousness, be read with interest. It clearly shows that there is nothing *specific* in the tumours following the inoculation of farcy; for nine similar tumours appeared during the progress of this disease. The symptoms form a suitable illustration of Amussat's correctness in attributing the fatal termination of the operation for imperforate anus, performed in the ordinary way, to absorption of fæces by the sides of the incision.

The *chronic* form of glanders next occupies our author's attention. Previously to reporting the cases published by Dr. Elliotson and Mr. Hardwicke, (the only examples of the affection in the human subject hitherto recorded,) he enters into a minute detail of the symptoms and lesions characteristic of the disease in the horse. This chapter will especially repay the trouble of perusal of those who take an interest in comparative pathology. It manifestly proves that chronic glanders is not, as Dupuy and others have maintained, a tuberculous affection. A certain degree of doubt unquestionably hangs over these cases, as respects the really glanderous nature of the disease. It is true the general character of the phenomena observed during life, as well as the ascertained appearances on dissection, argue in favour of its specific character; but, while ignorant of the anatomical state of the nares, we want the materials for coming to a positive conclusion. Again, of the seven cases drawn from French and German sources, and adduced as examples of *chronic farcy*, we confess ourselves unable to select one, the perusal of which carries with it conviction of its fair claim to such title. M. Rayer's argumentation in their behalf is acute and well sustained; but he at last admits the doubtfulness of their nature, and esteems them chiefly valuable (and herein we heartily concur with him,) as furnishing materials which may aid future research.

On the whole, we consider this essay a valuable production: it contains a clear, full, and impartial analysis of previous writings on the subject; furnishes us with a minute description of the structure of the bullæ and pustules; together with the results of microscopic examination of the various secreted fluids, and details of the only perfect autopsy hitherto published. The author argues with judgment on the question of transmission by inoculation, by contact, and by infection through the medium of the air; and gives us probably the best description that exists of the glanderous lesions in the horse. M. Rayer is the only writer on human glanders who appears to have fitted himself for its study by a diligent investigation of the disease in the solipeda; and, we think, he establishes the differential diagnosis of the disease, as it occurs in man, with greater accuracy than had hitherto been attained.

The treatise is illustrated by two well-executed quarto plates, containing sixteen coloured figures, representing the various morbid appearances produced by acute glanders in the human subject.

